# Cellular adaptation to oxygen deficiency beyond the Nobel award

**DOI:** 10.1038/s41467-020-14469-9

**Published:** 2020-01-30

**Authors:** José López-Barneo, M. Celeste Simon

**Affiliations:** 1 Instituto de Biomedicina de Sevilla (IBiS), Hospital Universitario Virgen del Rocío/CSIC/Universidad de Sevilla, 41013 Seville, Spain; 2 Departamento de Fisiología Médica y Biofísica, Facultad de Medicina, Universidad de Sevilla, 41009 Seville, Spain; 3 Centro de Investigación Biomédica en Red sobre Enfermedades Neurodegenerativas (CIBERNED), 41013 Seville, Spain; 4 Abramson Family Cancer Research Institute, University of Pennsylvania Perelman School of Medicine, Philadelphia, PA 19104-6160 USA; 5 Department of Cell and Developmental Biology, University of Pennsylvania Perelman School of Medicine, Philadelphia, PA 19104-6160 USA

**Keywords:** Cancer, Physiology

## Abstract

Understanding the cellular adaptation to oxygen deficiency -hypoxia- has a profound impact on our knowledge of the pathogenesis of several diseases. The elucidation of the molecular machinery that regulates response to hypoxia has been awarded the Nobel Prize in Physiology or Medicine.

The enrichment of Earth’s atmosphere in molecular oxygen (O_2_) by photosynthesis over the past billion years determined the appearance of sophisticated multicellular organisms, which led to the evolution of mammals and mankind. Our cells, in particular neurons and muscle, need O_2_ to extract the energy necessary to maintain essential vital functions from nutrients. This O_2_ is obtained from ambient air and transported to cells. Reduced O_2_ levels—or hypoxia—can happen systemically, due either to decreased atmospheric O_2_ or impairment of gas exchange in the lungs or locally in tissues and cells. Hypoxia, even if lasting only a few minutes, can produce irreversible cellular damage, as occurs in heart attacks or strokes. Therefore, both cellular adaptive biochemical responses to sustained hypoxia (hours or days) and organismal reflexes to acutely (in seconds) adapt to systemic hypoxia have evolved to compensate for the lack of O_2_ in mammals^20^. However, how cells/organisms detect O_2_ deficiency and the underlying molecular mechanisms are questions that have begun to be clarified in the past three decades. These advances have been recognized in 2019 with the Nobel Prize in Physiology or Medicine awarded to William G. Kaelin, Peter J. Ratcliffe, and Gregg L. Semenza.

## O_2_-regulated gene expression

The seminal discoveries originated in the laboratories of Semenza and Ratcliffe in the early 1990s who independently studied regulatory regions in the gene encoding erythropoietin (EPO), a hematopoietic growth factor synthesized in the kidney and liver that stimulates production of red blood cells in bone marrow. They identified a DNA sequence, for which they coined the term “hypoxia response element” (HRE), that was also present in other genes—such as those coding for vascular endothelial growth factor (*VEGF*) and multiple glycolytic enzymes—also transcriptionally regulated by hypoxia. Insertion of HRE sequences into regulatory regions of unrelated genes conferred O_2_ sensitivity^2–4^. These findings indicated that modulation of transcription by hypoxia is a generalized adaptive cellular response, affecting a broad array of targets (now >2500!) beyond those involved in erythropoiesis or angiogenesis. Culmination of this innovative work was the biochemical purification in Semenza’s laboratory of a protein induced by hypoxia (hypoxia-inducible factor (HIF)-1) able to bind to HREs. HIF-1 is actually a heterodimer with a constitutive β-subunit (HIF-1β) and an α-subunit (HIF-1α) regulated in an O_2_-dependent manner^5^. Two other isoforms of HIF (HIF-2α and HIF-3α) have been identified. HIF-2α, with a more restricted tissue distribution than HIF-1α, appears to be indispensable for the function of the EPO system^6^ or the carotid arterial chemoreceptors^7^ (see below).

**Fig. 1:**
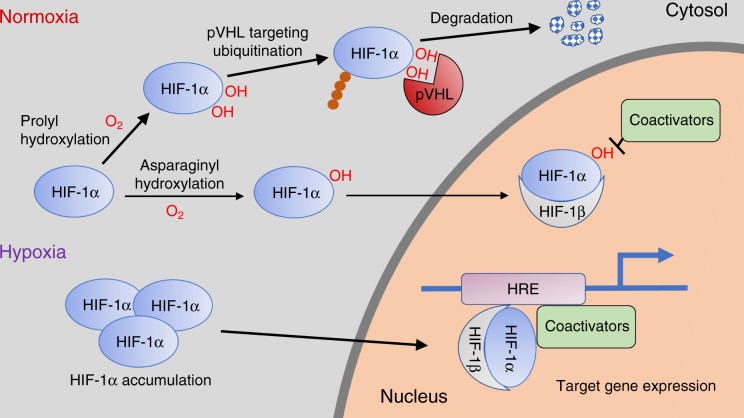
Scheme of the O_2_-sensitive HIF–PHD–pVHL signaling pathway. See text.

An understanding of how HIF is regulated by O_2_ levels came from Kaelin’s laboratory who were investigating highly vascularized kidney tumors generated by mutations in the von Hippel-Lindau (*VHL*) gene. They demonstrated that pVHL (the protein produced by *VHL*) is part of a ubiquitin ligase complex, involved in labeling of proteins for degradation, and that *VHL* deficiency resulted in constitutive expression of HIF-dependent genes even in the presence of normal levels of O_2_—normoxia. Among the most upregulated genes was *VEGF*, which explained the rich vascularization of tumors in the VHL syndrome^8^. Working independently, the Kaelin and Ratcliffe groups showed that pVHL targets HIF for its degradation by proteasomes and that HIF hydroxylation in specific proline residues is required for pVHL recognition. The link with O_2_ came after the discovery by these scientists (as well as by Richard Bruick and Steve McKnight of the University of Texas) of a family of prolyl hydroxylases (PHDs) responsible for HIF hydroxylation^9–11^. Unlike other prolyl hydroxylases, the HIF PHDs require relatively high concentration—tension—of O_2_, and in this manner, their dynamic range of activity occurs within physiological variations of O_2_ tension in organs and tissues. In normoxic conditions, with abundant O_2_ availability, the activity of PHDs is high and HIF-1α level is low, because hydroxylated HIF is rapidly targeted for degradation. In hypoxia, the shortage of O_2_ results in accumulation of non-hydroxylated—and non-degradable—HIF-1α, which after dimerization with HIF-1β binds to HREs in the regulatory regions of O_2_-sensitive genes (Fig. 1). An additional level of O_2_-dependent control of HIF transcriptional activity, originally identified in Semenza’s laboratory, is the factor inhibiting HIF, an asparaginyl hydroxylase that utilizes O_2_ as a substrate to hydroxylate asparagine residues, thereby inhibiting HIF-1α interaction with transcriptional coactivators^12^ (Fig. 1).

The work of William G. Kaelin, Peter J. Ratcliffe, and Gregg L. Semenza has been expanded by the collective scientific endeavor of this field, growing our understanding of O_2_ sensing and adaptation and leading the way toward medical applications. Given its ubiquitous localization, HIF functions as a master regulator of the expression of several thousand genes coding for a broad variety of growth factors, enzymes, transcription factors, cytokines, hormones, receptors, solute transporters, ion channels, etc. that participate in almost every cell function—or dysfunction. A myriad of publications has shown the importance of the HIF–PHD–pVHL system in essential processes, such as development and adult tissue/organ regeneration, cellular reprogramming, and stem cell biology. HIF also participates in the pathogenesis of highly prevalent lung and cardiovascular diseases, neurodegeneration, and inflammation, among others. However, it is in the field of cancer biology where the HIF–PHD–pVHL pathway has possibly had a greatest impact^13^. HIF is strongly upregulated in most tumors because malignant cells normally develop and grow in a hypoxic microenvironment. In addition to the activation of tumor angiogenesis and cell proliferation, HIF is responsible for the anerobic shift and increased uptake of glucose and its catabolism to lactate characteristic of cancer cells. In consequence, the HIF–PHD–pVHL pathway has pleiotropic effects on tumor growth and invasion as well as resistance to therapy^13^. In this regard, a pharmacological milestone in the short term may be the translation of direct and indirect HIF inhibitors as well as other agents modulating the “hypoxia response” into clinical practice^1^. These drugs, already in clinical trial, may have immediate use for the treatment of cancer as first-line treatment or in association with other anticancer drugs^14^. On the other hand, PHD inhibitors are also promising pharmacological tools for EPO induction to treat same forms of anemia^15^.

## New O_2_-regulated signaling pathways as chromatin and beyond

Elucidation of the O_2_-dependent HIF–PHD–pVHL signaling pathway has paved the way for a steady growth of the biology of O_2_ sensing, which is expected to continue in upcoming years. The discovery of new O_2_-dependent enzymes that, as PHDs, have low O_2_ affinity and are modulated over a wide range of O_2_ tensions, is further increasing the biomedical relevance of the study of the “hypoxic response”. Members of this group include several histone lysine demethylases, which regulate chromatin structure, and amino-terminal cysteine dioxygenases, which modify multiple relevant proteins posttranscriptionally.

It has been appreciated for some time that hypoxia increases histone methylation, but whether this reflects direct effects on histone demethylases was previously unclear. Recent reports indicate that several histone lysine demethylases (KDMs), specifically KDM5A and KDM6A/UTX, are responsible for a rapid induction of methylation of specific histones in hypoxic cells^16,17^. Interestingly, genomic locations of these methylated histones at a subset of hypoxia-inducible genes (e.g., *BNIP3L*, *KLF10*) predict a cell’s response to transient hypoxia several hours later, completely independent of HIF. Increased histone modifications associated with active gene transcription through KDM enzyme inhibition early during O_2_ deprivation could therefore co-ordinate subsequent transcriptional changes. Of note, hypoxia also increases the production of lactate by glycolysis, where lactate-derived lactylation of histone lysine residues also serves as an epigenetic modification that directly influences gene expression in O_2_-limited cells^18^.

Thiol oxidases, such as amino-terminal cysteine dioxygenases, also serve as O_2_ sensors regulating the stability of proteins like the angiogenic cytokine interleukin-32 or those involved in G protein signaling^19^. Here proteins targeted to the N-degron pathway in normoxia reversibly accumulate when the cysteine dioxygenase becomes substrate limited during O_2_ starvation. Moreover, similar dioxygenases are detected in plants and are likely to be conserved in a variety of multicellular eukaryotes. Such enzymatic oxygen sensors operate physiologically over a shorter time scale than transcriptional responses transduced by the HIF–PHD–pVHL system. The pathophysiological significance of these new O_2_-dependent enzymes and whether they also function in concert with the HIF pathway are challenging topics for future research.

## Acute systemic oxygen sensing

Sudden exposure to hypoxia, as occurs when moving to high altitude, elicits in mammals a cohort of acute organismal homeostatic responses—hyperventilation, tachycardia, or systemic vasodilation—that in few seconds increase O_2_ uptake and its distribution to multiple deprived tissues. These life-saving rapid reflexes are mediated by O_2_-sensitive chemoreceptor cells located in specialized sensory tissues/organs. Prototypical acutely O_2_-sensing organs are the carotid bodies (CBs), bilaterally connected by afferent nerve fibers with the brain respiratory and autonomic centers^20^. Dysfunction of the CBs has important medical consequences related to respiratory depression, hypertension, or the overactivation of sympathetic nervous system characteristic of patients with sleep apnea, heart failure, and insulin resistance. The molecular mechanisms underlying acute O_2_ sensing by chemoreceptor cells has remained elusive. However, recent work in CB glomus cells suggests that constitutive HIF-2α-dependent expression of atypical mitochondrial subunit isoforms renders cytochrome C oxidase in these cells highly sensitive to lowering O_2_ tension. Slowdown of the electron transport chain in hypoxia leads to accumulation of NADH and H_2_O_2_ of mitochondrial origin, which in turn modulate membrane ion channels to induce depolarization and transmitter release^7,20^. The requirement of HIF-2α for correct functioning of the CB O_2_-sensing pathway links acute and chronic adaptive responses to hypoxia.

This year’s Nobel Award has emphasized the value of living in an oxygenated world, adaptation to stressful conditions of hypoxia, and severe medical consequences of O_2_ deprivation. It is therefore a fundamental contradiction how much we disregard the health of our oceans and forests, where this precious gas is produced and to which we owe our lives.
