# Publisher Correction: Cellular adaptation to oxygen deficiency beyond the Nobel award

**DOI:** 10.1038/s41467-020-15018-0

**Published:** 2020-02-25

**Authors:** José López-Barneo, M. Celeste Simon

**Affiliations:** 1 Instituto de Biomedicina de Sevilla (IBiS), Hospital Universitario Virgen del Rocío/CSIC/Universidad de Sevilla, 41013 Seville, Spain; 2 Departamento de Fisiología Médica y Biofísica, Facultad de Medicina, Universidad de Sevilla, 41009 Seville, Spain; 3 Centro de Investigación Biomédica en Red sobre Enfermedades Neurodegenerativas (CIBERNED), 41013 Seville, Spain; 4 Abramson Family Cancer Research Institute, University of Pennsylvania Perelman School of Medicine, Philadelphia, PA 19104-6160 USA; 5 Department of Cell and Developmental Biology, University of Pennsylvania Perelman School of Medicine, Philadelphia, PA 19104-6160 USA

**Keywords:** Cancer, Physiology

Correction to: *Nature Communications* 10.1038/s41467-020-14469-9, published online 30 January 2020.

The original version of this Article contained an error in the first paragraph. Following the sentence ‘Therefore, both cellular adaptive biochemical responses to sustained hypoxia (hours or days) and organismal reflexes to acutely (in seconds) adapt to systemic hypoxia have evolved to compensate for the lack of O_2_ in mammals.’ ref. ^20^ was incorrectly cited instead of ref. ^1^. This has been corrected in both the PDF and HTML versions of the Article.

